# Assessing Eating Behaviour Using Upper Limb Mounted Motion Sensors: A Systematic Review

**DOI:** 10.3390/nu11051168

**Published:** 2019-05-24

**Authors:** Hamid Heydarian, Marc Adam, Tracy Burrows, Clare Collins, Megan E. Rollo

**Affiliations:** 1 School of Electrical Engineering and Computing, Faculty of Engineering and Built Environment, The University of Newcastle, Callaghan, NSW 2308, Australia; hamid.heydarian@uon.edu.au (H.H.); marc.adam@newcastle.edu.au (M.A.); 2 Priority Research Centre for Physical Activity and Nutrition, The University of Newcastle, Callaghan, NSW 2308, Australia; tracy.burrows@newcastle.edu.au (T.B.); clare.collins@newcastle.edu.au (C.C.); 3 School of Health Sciences, Faculty of Health and Medicine, The University of Newcastle, Callaghan, NSW 2308, Australia

**Keywords:** eating activity detection, hand-to-mouth movement, wrist-mounted motion tracking sensor, accelerometer, gyroscope

## Abstract

Wearable motion tracking sensors are now widely used to monitor physical activity, and have recently gained more attention in dietary monitoring research. The aim of this review is to synthesise research to date that utilises upper limb motion tracking sensors, either individually or in combination with other technologies (e.g., cameras, microphones), to objectively assess eating behaviour. Eleven electronic databases were searched in January 2019, and 653 distinct records were obtained. Including 10 studies found in backward and forward searches, a total of 69 studies met the inclusion criteria, with 28 published since 2017. Fifty studies were conducted exclusively in laboratory settings, 13 exclusively in free-living settings, and three in both settings. The most commonly used motion sensor was an accelerometer (64) worn on the wrist (60) or lower arm (5), while in most studies (45), accelerometers were used in combination with gyroscopes. Twenty-six studies used commercial-grade smartwatches or fitness bands, 11 used professional grade devices, and 32 used standalone sensor chipsets. The most used machine learning approaches were Support Vector Machine (SVM, *n* = 21), Random Forest (*n* = 19), Decision Tree (*n* = 16), Hidden Markov Model (HMM, *n* = 10) algorithms, and from 2017 Deep Learning (*n* = 5). While comparisons of the detection models are not valid due to the use of different datasets, the models that consider the sequential context of data across time, such as HMM and Deep Learning, show promising results for eating activity detection. We discuss opportunities for future research and emerging applications in the context of dietary assessment and monitoring.

## 1. Introduction

Recent advances in the accuracy and accessibility of wearable sensing technology (e.g., commercial inertial sensors, fitness bands, and smart watches) has allowed researchers and practitioners to utilise motion sensors mounted on the upper limbs (i.e., lower arm/wrist, upper arm) to assess dietary intake and eating behaviour in both laboratory and free-living conditions. Inertial sensors such as accelerometers (e.g., [1,2]) and gyroscopes (e.g., [3,4]), as well as proximity sensors (e.g., radio-frequency identification (RFID) [5,6]), can be used to detect and quantify characteristic hand-to-mouth gestures associated with food and beverage consumption. As such, compared to other types and/or positioning of sensors (e.g., mounted to a user’s neck or head), this technology offers advantages in terms of detecting the timing and amounts of eating behaviour in an unobtrusive, accessible, and affordable way that yields high levels of technology acceptance [7,8,9]. Disadvantages, including the limited ability to detect brief snacks and the type and amounts of food being consumed [10], can be addressed by combining these sensors with other active (e.g., self-reporting with a food record or recall) and passive capture methods (e.g., microphone, video). In this vein, one can use the data gained from upper limb motion sensors to (1) improve and complement traditional dietary assessment methods [11] (e.g., by triggering reminders to actively take a photo when an eating occasion is detected), and (2) to support the delivery of dietary behaviour change interventions, for instance by capturing characteristic hand-to-mouth movements (e.g., [1,12]).

Overall, the field of wrist-mounted motion tracking sensors for the measurement of eating behaviour has evolved rapidly over the past decade. In 2012, Dong and colleagues [13] used a relatively expensive device (about US $2000 per device) called the InertiaCube3 (tri-axial accelerometer, tri-axial gyroscope, and tri-axial magnetometer) that was wired to a separate reader device. Within only a few years, the price of these sensors dropped to less than US $200. At the same time, sensors have now substantially reduced in size, operate wirelessly, and are powered by rechargeable batteries (e.g., [9,14]). High-quality motion tracking sensors are available within off-the-shelf, commercial smart watches that can be purchased at a fraction of the price of earlier devices and by the general population, not just researchers. Fuelled by these technological advances, and with wearable motion tracking devices experiencing rapid growth in areas such as fitness [15], the field of monitoring and assessing eating behaviour and dietary intake using these technologies is evolving. Another factor contributing to the rapid proliferation of motion tracking devices is their high level of technology acceptance; such devices have become increasingly culturally acceptable and unobtrusive to wear [7,8,9,16]. Combined with machine learning methods, the collection of movement data from wrist-mounted motion tracking sensors can be used to extract meaningful information about a person’s daily activities (e.g., eating behaviour and physical activity) in a continuous, scalable, and discreet way [2,17].

Several recent reviews have explored how wearable sensors have been applied to the field of nutrition for assessment or monitoring of eating behaviour and/or dietary intake [10,15,16,18,19,20]. However, previous reviews have focused on a wider range of wearable sensors (e.g., camera, microphone) located on various parts of the body (e.g., ear, temple, torso) and included a range of eating-related activities [10,16,18,19] (e.g., chewing, swallowing), or focused on smoking [15]. Hassannejad and colleagues [18] reviewed two main approaches used to try and automate dietary monitoring. The first approach was to automatically extract information on food content based on image analysis and the second approach was to extract the information sourcing data from wearable sensors to detect eating behaviour. Kalantarian and colleagues [10] provided a general overview of dietary monitoring technology (e.g., acoustic, image, inertial, and manual food diaries). Prioleau and colleagues [16] focused specifically on wearable sensors such as cameras, microphones, and motion sensors placed on different body locations (e.g., ear, mouth, neck, upper limb). Vu and colleagues [19] provided an overview of data analytic and sensing platforms for wearable food intake monitoring, covering a wide range of systems including acoustic, inertial, muscle activity, proximity, and visual sensors, rather than focusing on a specific type of wearable sensors. Parate and colleagues [15] reviewed approaches designed for detecting eating and smoking behaviours where hand gestures are involved. Doulah and colleagues [20] conducted a systematic review of studies focusing on estimating dietary energy intake (i.e., amount of consumption, energy density, and food recognition), covering a range of image-based approaches (e.g., depth, smartphone, or wearable cameras) and wearable sensors (e.g., chewing sound, jaw motion, or wrist motion sensors). However, to the best of our knowledge, there is currently no systematic review of the current state of research available that specifically focuses on the use of upper limb-mounted motion tracking sensors for assessing eating behaviour.

This review differs from existing reviews in the area in that it includes a systematic search and thereby follows a rigorous process with two reviewers to provide an overview of the current state of research on upper limb-mounted motion sensors for assessing eating behavior across the 69 identified studies. Given the wide availability and affordability of upper limb-mounted motion sensors, an understanding of the study settings, sensor configurations, detection approaches, and eating behaviour assessment in the extant literature is important in order to progress research in this area and inform the application of these approaches in practice. Hence, the aim of the current review is to summarise the current evidence on use of upper limb-mounted motion sensors for assessing eating behaviour.

## 2. Materials and Methods

### 2.1. Definition of Common Terms

Table 1 provides an overview of the terms and definitions employed in the current review. Throughout this review, we use the term *motion sensor* to refer to wearable motion tracking or wearable motion detection sensors, unless specified otherwise. Wearable motion sensors are usually integrated into a tracking device mounted on the wrist or other parts of the upper limbs (e.g., activity tracker, fitness tracker, smart watch). The tracking device commonly consists of several different motion sensors such as inertial sensors and proximity sensors. A *proximity sensor* can detect the presence of nearby objects and therefore requires a separate sensing device. An *inertial sensor* can detect changes in linear or angular momentum. The two most widely-used inertial sensors are three-dimensional micromachined microelectromechanical systems (MEMS) accelerometers and gyroscopes. While the tri-axial *accelerometer* measures magnitude and direction of acceleration on X, Y and Z axes, the tri-axial *gyroscope* measures the rate of rotation on yaw, pitch, and roll axes. The studies across the field have used a variety of different terms to refer to the same concept. Action classes are the desired types of events to be detected through the artificial intelligence models. The action classes vary depending on the machine learning approach taken and the behaviour assessment outcomes expected. These classes need to be predefined with labels (i.e., tagged), and used in the process of data annotation to mark the events (e.g., using video cameras or self-report push buttons). The events are marked with the start time, end time, and a label (action class) that described what the event is about. An event may be marked with multiple labels (e.g., drinking, left hand).

### 2.2. Search Strategy

For the current review, we included studies that (1) used at least one wearable motion sensor, (2) that was mounted to the wrist, lower arm, or upper arm (referred to as the upper limb in this review), (3) for eating behaviour assessment or human activity detection, where one of the classified activities is eating or drinking. We explicitly also included studies that additionally employed other sensors on other parts of the body (e.g., cameras, microphones, scales). In order to identify studies that meet these criteria, we constructed the search string to include three parts (motion sensor, mounted to upper limb, eating behaviour assessment). The search string was then iteratively developed from a set of key studies that were identified in an initial search as well as from MeSH headings and consultation with a medical librarian. Using multiple combinations of search terms shown in Table A1 and Table A2 a comprehensive search was conducted to interrogate electronic archives across medical and health sciences as well as computing disciplines for studies published in English. In computing the ACM digital library, AIS electronic library (AISeL), IEEE Xplore, ScienceDirect, SpringerLink archives and in health sciences the CINAHL, MEDLINE, EMBASE, Ovid, Web of Science and Scopus archives, eleven in total were searched. In order to account for the breadth of publications in health and computing-focused outlets, the search covered peer-reviewed studies published in book chapters, journals, and full conference proceedings (excluding abstract-only/extended-abstract papers). Particularly in computing, studies are often published as full conference papers. The search terms combination was adapted to each electronic archive due to their limitation on search input. The search was conducted in January 2019 and backward and forward search was done after the included studies were identified.

The review protocol was registered with Prospero system (the CRD42018089493). The primary outcomes assess upper limb-mounted motion sensors and devices used to detect hand-to-mouth gestures associated with eating. This is to identify what types of sensors were used, how the sensors were combined or used together, and where on the upper limb they are mounted. The secondary outcomes assess the algorithms and techniques utilised to analyse the output of the sensors used on body for motion tracking associated with eating occasions, the environmental conditions under which the experiments were been conducted (e.g., setting, food items, serving vessels and eating utensils), and the characteristics of eating behaviour that were assessed (e.g., bite count, duration of eating, quantification of amounts, and type of the food eaten).

### 2.3. Selection Process

The results of the database search were imported into a web-based tool (Covidence [21]), duplicate items were identified and removed, and the rest of the studies were title- and abstract-screened by two of four independent reviewers (H.H., M.A., T.B., M.E.R.) to identify studies that potentially meet the inclusion criteria. The full text articles were then retrieved and assessed for eligibility by two of the four independent reviewers, with discrepancies resolved by discussion with a third independent reviewer who was not involved in assessing that particular study.

Following the selection of studies, two reviewers independently extracted relevant information using a custom-made data collection form; any discrepancies regarding this data were resolved by discussion with a third reviewer. Data from the selected studies were captured and summarised in Table 2 which was constructed for the purpose of this review. These were initially pilot tested with seven studies to ensure all data was extracted and appropriate. Due to the nature of this review evaluating the performance of technology, a risk of bias assessment was not deemed to be necessary/appropriate by the research team. Countries of data collection were categorised by economies according to a UN report [22].

## 3. Results

**Figure 1:**
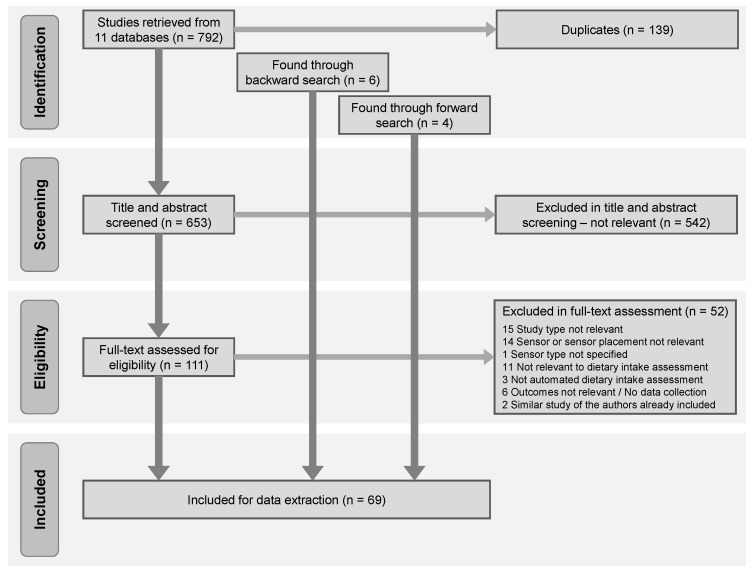
Flow diagram of article selection process in the systematic review.

In total, 792 studies were identified through the search strategy, after removing 139 duplicates, 653 studies were screened on title and abstract. Of these, 111 were full-text reviewed independently by two authors, with a third author coming in if consensus was needed. With six studies found through backward search and four studies found through forward search, 69 studies were included in the review (Figure 1). 

**Figure 2:**
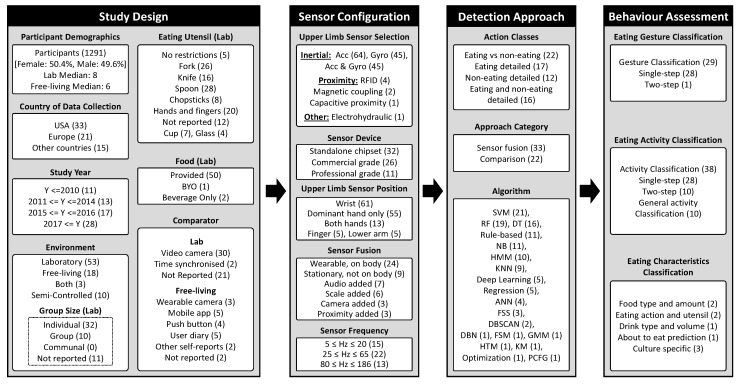
Conceptual framework of components for assessing eating behaviour with upper limb-mounted motion sensors.

This review provides a narrative synthesis of the findings from the included studies and uses these finding to structure a conceptual framework (Figure 2). In particular, we reviewed the selected studies to identify common components and implicit design choices that are involved in carrying out research in this area. We then synthesised this knowledge into a conceptual overview. The framework depicts an overview of the process of assessing eating behaviour using upper limb-mounted motion sensors and the different components involved in the process. Thereby, *study design* pertains to the environmental conditions that the participants experience as well as the requirements, instruments, and instructions for data collection process. In contrast, *sensor configuration* summarises the specific type, sampling frequency, and position of the employed motion sensor(s). These are the main components required to build a model to detect eating behaviour shown under *detection approach*. This process leads to identifying and assessing dietary behaviour which is depicted under *behaviour assessment*. The framework provides a structure for the synthesis and presentation of results in this review. Please note that some subcategories are not shown in Figure 2 because no studies were identified for them. For instance, none of the reviewed studies used sensor frequencies between 21 and 24 Hz.

### 3.1. Study Design

#### 3.1.1. Participant Demographics

The number of participants ranges from one (i.e., [8,24,28,31,36,44]) to 276 [52] (median: 8 in lab setting, 6 in free-living setting). The total number of participants who successfully participated in the experiments was 1291. Of the included studies that reported participant gender (*n* = 36, 52.2%), 50.4% of participants were female and 49.6% were male. According to the demographic data where social class was reported, the participants were commonly university students.

#### 3.1.2. Country of Data Collection

In all studies, all data collection was done in the country of the first author’s affiliation. Most studies were conducted in the US (*n* = 33, 47.8%), followed by Europe (*n* = 21, 30.4%). Singapore, South Korea, India, and Mexico had two studies each. Australia, Canada, and Japan hosted one study each. As it can be seen the data is mostly collected in high-income countries (94.2%). Only two studies were conducted in a lower-middle-income country (India). No study collected data in a low-income country.

#### 3.1.3. Study Year

Up to and including 2010, only 11 studies (15.9%) were published in this field, with 13 Studies (18.8%) published between years 2011 and 2014. Seventeen studies (24.6%) were published between years 2015 and 2016. Interestingly, 40.6% of the studies (28 studies) were published in 2017 or later.

#### 3.1.4. Environment

The majority of the studies were conducted exclusively in controlled laboratory settings (*n* = 50, 72.5%; e.g., [55,65]), followed by exclusively free-living settings (*n* = 15, 21.7%; e.g., [5,51]), with fewer being conducted in both settings (*n* = 3, 4.3%; [2,3,7]). One study (1.4%) did not report the environment setting. The three studies that considered laboratory as well as field data used the data collected in the laboratory for training the machine learning models and the data from the free-living environment for evaluating the model’s performance (e.g., [2,3,46]). The laboratory environment may affect the participant’s natural behaviour in the progress of experiment. Therefore, 10 studies (14.5%) conducted semi-controlled experiments in laboratory environments (i.e., [2,14,65]) or in a cafeteria, restaurant, or dining hall (i.e., [1,12,39,52,60,61,77]).

The laboratory environment commonly involved participants sitting individually (e.g., [25]) at a table or in a group (e.g., four people [1,39]) around a table recorded with video camera(s) to capture the eating session. In a study by Amft and Tröster [25], participants were instructed to perform non-eating tasks such as reading a newspaper (including turning pages), scratching their head, and answering a simulated mobile phone call. The leftover food from the participant’s meal could either be weighed throughout the experiment to keep track of food consumed [48] or at the end of the session to estimate the total amount of the food consumed [17,55]. However, few studies measured leftover food [17,39,48,52,55].

Studies in free-living environments commonly allowed participants to perform their daily activities during the day while wearing the sensor(s). The longer duration experiments involved more non-eating associated activities (e.g., driving, watching TV and working on a computer) than eating activities. Thomaz and colleagues [2] conducted an experiment in both settings. For the laboratory setting, the average duration of the data collection was 31 min which included 48% eating activities. In contrast, of the two experiments conducted in free-living conditions, one had an average duration of 6 h and included 6.7% eating activities while the other one was carried out over 31 days and included only 3.7% eating activities. Several studies indicate challenges associated with field data collection. In a free-living study by Dong and colleagues [41], data from ten out of a subsample of 30 individuals were discarded due to poor compliance with keeping manual records of activities (e.g., misinterpreting the instructions and starting/stopping recording for meals only). In a study by Sharma and colleagues [51], data collected from 10% of the 104 individuals were discarded because they failed to wait ten minutes between wearing the device and the first meal.

Among the 53 studies conducted in the laboratory, 32 studies (60.4%) asked participants to eat individually from a discrete plate (e.g., [26,65]), 10 studies (18.9%) were carried out in groups comprising between two to four people (e.g., [1,12]), and 11 studies (20.8%) did not report the group size. In group settings, participants were still provided with discrete plates of food and/or asked to self-serve on to their own individual plate. No experiment was reported asking the participants to share food from one or more plates (communal eating).

#### 3.1.5. Eating Utensils

The utensils most commonly used in laboratory experiments were spoons (*n* = 28, 52.8%), followed by forks (*n* = 26, 49.1%), knifes (*n* = 16, 30.2%), and chopsticks (*n* = 8, 15.1%). Five studies (9.4%) conducted in the laboratory applied no restriction on the type of eating utensils. Eating with hands or fingers was reported in 20 studies (37.7%) conducted in the laboratory. Twelve studies (22.6%) conducted in the laboratory did not report what utensils were used. The studies that reported drinking vessels used cups (*n* = 7, 13.2%) or glasses (*n* = 4, 7.5%). However, participants were served with yogurt in a mug in one study [24] and the use of a straw to drink beverages was reported in four studies [3,17,40,48]. Zhang and colleagues [17] reported that drinking from a straw for longer than 30 s produced unusual motion sensor data which was disregarded as a single gesture.

#### 3.1.6. Food

In the experiments conducted in free-living conditions the participants consumed their own food (*n* = 18, 26%). In contrast, food in the laboratory settings was commonly provided for participants by researchers. Only in one laboratory study [3], participants were asked to bring their own foods and drinks. One of the most reported foods eaten with a fork and knife in the experiments was lasagne (i.e., [2,23,24,25,26,65], *n* = 6). Rice (e.g., [6,35,42]) and soup (e.g., [35,40,53]) were commonly eaten with a spoon, whereas pizza (e.g., [12,40,48]) and bread (e.g., [12,43,48]) were commonly eaten with hands. Kim and colleagues [35] collected data from participants eating rice with both chopsticks and spoon.

Some of the other food items reported in laboratory settings were chips/fries, burger/sandwich, fruit, meat/steak, pasta, salad, vegetables, yoghurt, and snack foods (e.g., cake, candy, chocolate, ice-cream, popcorn). In addition, various beverages (e.g., coffee, juice, smoothie, soda, tea, and water) were provided for participants to drink while consuming food. Some studies (e.g., [1,12,48]) provided the participants with multiple food options so they could self-select amounts and types of food. These studies were usually conducted in a semi-controlled environment. In comparison, two laboratory studies (i.e., [32,64]) exclusively examined drinking behaviour. In one of these studies, Amft and colleagues [32] used nine different drink containers to investigate the recognition of container types and the volume of fluid consumed from the container.

The duration of an uninterrupted eating episode in a controlled environment depends on the number of hand-to-mouth gestures and chewing time, which is directly related to the food type. Sen and colleagues [7] observed that eating episodes ranged from 51 s for fruit to 19 min for rice.

#### 3.1.7. Comparator

To facilitate sensor data analysis, collected data must be annotated with differing labels to represent actions and events that occurred. The annotated data is then used to train the machine learning models and evaluate their performance. One approach for data annotation is to let participants self-report the investigated activities in real-time using a mobile app and/or a push-button technique (i.e., [5,6,14,37,45,54]). Further, some studies in free-living environments combined a push-button approach with a pen and paper diary (e.g., [5]) or an electronic food diary on a smartphone (e.g., [54]) completed by the participant. However, these commonly employed comparator techniques rely on participants to provide an accurate and complete record of activities. Hence, it is not possible to unambiguously establish ground truth. By contrast, for experiments conducted in laboratory settings, ground truth can be established by using objective observation instruments. This is commonly achieved through video cameras.

Of the 53 laboratory studies, 32 (60.4%) reported the comparator. Thirty (56.6%) used video recordings to establish ground truth (mostly surveillance video with one study using a wearable camera [42]), while the other two studies (3.8%) used different time synchronisation mechanisms (timestamps for predetermined tasks [56] or alarms to instigate drinking [64]). Of the 18 free-living studies, only two (11.1%) did not report the comparator. Five studies (27.8%) used a diary, five (27.8%) used a self-report mobile app, and four (22.2%) used a button on the wearable sensor device. Interestingly three studies (16.7%) used wearable camera to establish ground truth on the free-living environment and two studies (11.1%) used other self-report/self-recall approaches.

### 3.2. Sensor Configuration

#### 3.2.1. Sensor Selection on the Upper Limbs

The most commonly used motion sensors that were mounted on the upper limbs are (tri-axial) accelerometers (*n* = 64, 92.8%) and (tri-axial) gyroscopes (*n* = 45, 65.2%). Interestingly, all 45 studies that used a gyroscope also used an accelerometer. Seven studies (10.1%) used proximity sensors on the upper limbs. This includes RFID sensors (four studies; [5,6,37,45]), magnetic coupling sensors (two studies; [28,32]), and capacitive proximity sensor (one study, combined with accelerometer; [34]). One study [49] used electrohydraulic sensors. Additional proximity sensors mounted to the drinking vessel [29] or the eating utensils (fork, knife and cup) [36] were also reported. Amft and colleagues [32] used a magnetic coupling sensor where the field emitting sensor was attached to the shoulder while the receiver unit was attached to the wrist.

#### 3.2.2. Sensor Device

The majority of studies directly used standalone sensor chipsets rather than an integrated recording device (*n* = 32, 46.4%). Twenty-six studies (37.7%) used off-the-shelf, commercial-grade smartwatches or fitness bands, such as Microsoft Band and Pebble watch. Eleven studies (15.9%) used professional grade devices with embedded sensors such as Shimmer and XSens. In recent years, more studies have tended to use off-the-shelf, commercial-grade smartwatches or fitness bands and less studies employed standalone sensor chipsets. One study [41] used the accelerometer and gyroscope embedded in a smartphone (iPhone 4) mounted on the forearm (wrist). However, a smartphone was used in another study [2] to conduct a pilot formative experiment before collecting data using accelerometer and gyroscope sensor modules. One study [28] used a professional grade device (Xsens) as well as a standalone sensor chipset.

#### 3.2.3. Sensor Position on Upper Limbs

Sixty-one studies (88.4%) used at least one motion sensor on the wrist and five studies (7.2%) reported at least one motion sensor mounted to the lower arm. Four studies [44,58,63,74] used an inertial sensor on a finger in addition to the wrist, while another study [9] only used an accelerometer worn on an index finger. Five studies (7.2%; [23,24,25,26,28]) used motion sensors on the upper arm as well as wrist or lower arm. One study [36] used motion sensors only on utensils (fork, knife and cup), and another study [49] used electro-hydraulic sensors on both hands. Fifty-five studies (79.7%) used the motion sensors only on the dominant eating hand, while thirteen studies (18.8%) used the motion sensors on both hands.

#### 3.2.4. Sensor Fusion

Thirty-three studies (47.8%) combined upper limb-mounted motion sensors with other sensors on different parts of the body or in the environment. Twenty-four of these studies (34.8%) used different types of sensors on or attached to the participants’ body (i.e., torso, chest, upper back, head, jaw, throat, ear, foot) or in participants’ pocket in addition to their upper limbs. The other studies (n=9, 13.0%) used sensors placed in the participants’ environment (e.g., camera, scale, and proximity). For example, Amft and Tröster [25] used (inertial) motion sensors including accelerometer, gyroscope, and compass on lower arm, upper arm, and upper back, all attached onto a jacket to detect movement activities. Further, they used an ear microphone (electret miniature condenser microphone) to detect chewing activities as well as a stethoscope microphone mounted to the hyoid and an electromyogram (EMG) mounted to the infra-hyoid throat to detect swallowing activities. Six studies (8.7%) used scales to measure the weight of food consumed throughout the experiment (i.e., [17,39,48,52,55,64]). Further, several studies combined motion sensor data with audio (*n* = 7, 10.1%; [24,25,48,50,57,59,62]) or video camera recordings (*n* = 3, 4.1%; [31,72,74]) to detect eating behaviour. For instance, Mirtchouk and colleagues [48] combined accelerometer data from each participant’s both wrists and head with audio data recorded from a pocket audio recorder. Garcia-Ceja and colleagues [59] combined accelerometer data with audio data collected from a smartphone placed on a table in the same room as the participant to record environmental sound.

#### 3.2.5. Sensor Sampling Frequency

Sensor sample rate (frequency) is the number of data items the sensor collects per second. Forty-nine studies (71%) reported the sample rate, with frequencies for the wrist-mounted motion sensors, ranging from 5 Hz [58] to 186 Hz [64]. Among these, 15 (21.7%) used a frequency of lower or equal to 20 Hz, 22 (31.9%) used a frequency between 25 Hz and 65 Hz, and 13 (18.8%) used a frequency of 80 Hz or more. The median sampling frequency was 50 Hz. Five studies [1,12,39,51,52] used both an accelerometer and a gyroscope with a 15 Hz sample rate frequency, whereas three studies [23,24,25] also used both an accelerometer and a gyroscope but with a higher rate of 100Hz.

### 3.3. Detection Approach

This section discusses the categories that eating detection approaches fall into, algorithms used to build detection approaches, and types of gestures and activities defined for prediction, referred to in this review as action classes. Detection approaches commonly involved three consecutive stages: pre-processing, feature extraction, and building an eating action detection model.

#### 3.3.1. Action Classes

The action classes at the simplest level (binary) were eating and non-eating actions (*n* = 22, 31.9%). Thereby, we can distinguish between *gesture detection* (characteristic low-level actions) and *activity detection* (high-level actions). In 17 studies (24.6%) only eating associated actions were detailed to subcategories. In 12 studies (17.4%) only non-eating associated actions were detailed to subcategories. In 16 studies (23.2%) both eating and non-eating associated actions were subcategorised. Kim and colleagues [35], defined the classes to detect the utensil type in addition to the eating action. Amft and colleagues [32] defined nine different drinking vessels as the action classes for the purpose of container type and fluid level recognition.

#### 3.3.2. Approach Category

We can identify two approaches for eating behaviour assessment: eating gesture detection and eating activity detection. At the lower level, in *eating gesture detection* (*n* = 29, 42%), the aim is to detect characteristic eating gestures that are the building blocks of eating occasions while in *eating activity detection* (*n* = 38, 55.1%), the aim is to detect the occasions when the participant was eating. For instance, a period of time can be categorised as an eating occasion when at least a certain number of eating gestures occur in a row. There are mainly two different approaches to implement an eating activity detection solution single-step and two-step. In the *single-step* approach (*n* = 28, 40.6%; e.g., [6,42,47]), the eating detection model is trained on pre-processed motion data with the aim of detecting the pre-defined activities (e.g., eating events versus non-eating events). In the *two-step* approach, two different models are consecutively employed where typically the first model is responsible to detect the desired hand gestures using pre-processed data as input. The model at the second step uses the output of the first step as its input to detect the desired activities (*n* = 10, 14.5%; e.g., [7,51,60]).

Further, sensor fusion methods may also be utilised in the two above-mentioned approaches. In the *fusion* approach (e.g., [6,59,72]), researchers collect data using multiple sensors on different body parts or combine wearable and stationary sensors, as opposed to collecting data from sensor(s) mounted on one position on body. In this approach typically multiple classifiers are used where the outputs of the classifiers will be aggregated to detect desired activities based on action classes.

#### 3.3.3. Algorithm

Table 3 provides an overview of the machine learning algorithms and detection approaches used in the reviewed studies. It also demonstrates the experiments conducted to compare the performance of the machine learning algorithms. Thereby, in order to avoid repetitions, each comparison study is only listed once, namely for the algorithm where the comparison yielded the best performance. Twenty-two studies (31.9%) compared the performance of different algorithms. Naive Bayes was used for benchmarking where multiple algorithms were compared.

### 3.4. Eating Behaviour Assessment

#### 3.4.1. Eating Gesture Classification

The aim of eating gesture classification is to detect characteristic gestures involved in ingestive behaviours (e.g., hand-to-mouth gestures). Such gestures are produced when an individual picks up food and moves it towards his/her mouth (hand-to-mouth movements, with or without utensils). Twenty-nine studies (42%) targeted only different aspects of eating gesture classification. Detecting eating gestures is often achieved with a single-step classification technique. However, researchers in [67] used two steps for eating gesture classification. They used a sliding window technique to first detect stationary periods, where the participants were more likely to eat, and the model then detected eating-associated gestures in the next step.

#### 3.4.2. Eating Activity Classification

Twenty-eight studies (40.6%) used a direct detection approach for eating activity classification, i.e., detecting eating activities without detecting eating gestures first (e.g., [6,33,65]). Ten studies (14.5%) built eating gesture detection models as the first step to then detect eating activities in the second step (e.g., [2,51,60]). In other words, these studies employed a two-step detection approach, where the eating gestures detected in the first step are used to build a model in the second step to differentiate eating and non-eating activities (e.g., brushing teeth, combing hair, talking on the phone, walking, watching TV, and writing). Ten studies (14.5%) conducted general activity detection where eating activities were included in the data collection process along with a range of other activities and then classified in the activity detection approach (e.g., ambient assisted living).

#### 3.4.3. Eating Characteristics Classification

In addition to detecting eating gestures and eating activities, six studies (8.7%) aimed to detect further characteristics of eating behaviour, i.e., food type and amount detection (*n* = 2, 2.9%; [38,48]), eating action and utensil detection (*n* = 2, 2.9%; [35,71]), drink type and volume detection (*n* = 1, 1.4%; [64]), and also about-to-eat and time until the next eating event prediction (*n* = 1, 1.4%; [50]). Mirtchouk and colleagues [48] investigated food type detection and amount consumed. Kim and colleagues [35] detected different types utensils (i.e., chopsticks, hand, spoon) as well as eating and non-eating gestures such as stirring, picking up rice, and using tissue. Rahman and colleagues [50] designed a system to predict the next eating occasion. Soubam and colleagues [64] detected drink type and volume in addition to eating and drinking gesture detection. Three studies (4.3%, [35,38,71]) specifically explored the Asian eating style. Cho and Choi [71] focused on eating action and utensil detection specifically for Asian-style food intake pattern estimation (chopsticks vs. spoon).

## 4. Discussion

The current review set out to synthesise existing research that describes the use of the upper limb-mounted motion sensors for assessing eating behaviour. Based on the 69 studies identified in our search, we are able to document the current body of research in the detection of *eating activities* (e.g., drinking, eating) and individual *eating gestures* (e.g., specific hand-to-mouth movements). To this date, most studies were carried out in laboratory conditions with university student (young healthy adults), with limited application in free-living settings or in diverse publication groups. Devices used were predominantly accelerometers in combination with gyroscopes worn on the wrist of the dominant hand, and the focus so far lied on distinguishing eating from non-eating activities.

### 4.1. Research Environments and Ground Truth

The conditions and restrictions of the research environments have implications for different aspects of the eating detection approach; these are important considerations, given that the majority of the included studies were conducted in a laboratory setting. As a result, the accuracy achieved in testing models with data collected from the free-living settings may be lower compared to models trained and tested on the laboratory data. However, few studies collected data from free-living environments for evaluation purposes. Using data collected from free-living environment for training purposes will likely help improve the performance of detection models in less controlled settings. Future studies may overcome this issue by combining laboratory and free-living data approaches in a multi-stage approach to study design. Few studies have combined lab and free-living data (e.g., [2,3,46]) to date. For instance, Ye and colleagues [43] first trained a model in a laboratory study. In a follow-up study [54], they then used buttons on a smart watch (Pebble) and an app (Evernote) to confirm or reject detected eating occasions when testing the model in free-living setting.

To implement a machine learning model to automatically identify eating gestures, accurate data containing the target activities or the “ground truth” is required. The machine learning model then learns from this data and can later be used for automated eating activity detection. Objective ground truth tools (e.g., video cameras) are more practical in laboratory settings. Such controlled settings are imperative to increase the accuracy of data annotation which is crucial for building and evaluating classifiers. Only a few studies in free-living settings have used passive capture of video as the measure of ground truth (e.g., [31]). In contrast, most studies in free-living settings rely on participants self-reporting the target activities by using tools such as diaries or push buttons on a device [5]. However, even for data for which a video recording exists, the annotation of the exact start and end times of eating gestures can be ambiguous, which in turn may affect a model’s accuracy. Difficulties could include the assessment of the exact moment when the hand-to-mouth movement starts and when the hand returns to an idle state, synchronisation across multiple devices or sensors (e.g., wrist sensor for gesture capture with video of eating activity; [48]), obstruction of ground truth measurement due to unrelated movements, people, or objects in certain settings such as communal eating.

### 4.2. Eating Context and Population Groups

The characteristics of eating movements, and the volume of food consumed, may change in different contexts (e.g., when the participant is stressed, walking, or working). However, the impact of context on the accuracy of automatically detecting eating gestures is yet to be explored. Snacking or in-between meal eating has widely been disregarded in the surveyed studies, possibly because it is difficult to detect sporadic eating-associated with hand-to-mouth movements in a free-living setting and it could easily be confused with other movements. Eating behaviour assessment is often based on a two-step approach that links individual eating gestures to timeframes of eating activities. Further, the majority of lab studies provided food to participants, often with a limited variety in type, which is in contrast to the wide variety of food available in free-living settings. Further, the majority of studies in laboratories were carried out with university students, therefore the movement data may not be representative for other population segments (e.g., elderly, young children, clinical populations). Another important contextual factor is eating culture. For instance, Cho and Choi [71] and Kim and colleagues [35,38] specifically explored the Asian eating style and found that hand movements associated with eating with a spoon are characteristically different from those associated with eating with chopsticks. Different cultural aspects of eating behaviour have been overlooked in the literature. For instance, at this stage there are no studies that consider data from communal and shared plate eating (e.g., with servings from a shared dish [79]). Abkenar and colleagues [62] investigated a context where two participants shared a meal together, yet this did not involve a shared dish. Communal eating is an important form of eating in many cultures (e.g., [79,80,81]). Further, there has been no study that has considered using upper limb motion sensors for detecting eating behaviour of individuals from low and lower-middle income countries. All of the settings mentioned will likely include additional challenges due to characteristic hand movements associated with serving food from communal dishes to individual serving vessels.

### 4.3. Advanced Models and Deep Learning

Machine learning algorithms employed to detect eating behaviour are distinguished by whether and how they consider the sequential context. Classifiers such as K-nearest neighbours (KNN) or support vector machine (SVM) do not explicitly utilise the sequential aspect of data. By contrast, classifiers such as Hidden Markov Model (HMM) and Recurrent Neural Networks (RNN) take into account the sequential context, using previous states of data to predict the current state of data. The latter types have gained more attention recently ([61,68,71,73,75]). In the current review most studies used approaches that do not model the sequential context of data across time (e.g., 21 SVM, 19 Fandom Forest, 16 Decision Tree, 9 KNN) while recently more studies have considered the sequential context (10 HMM, 4 RNN). These recent models have shown promising results. For instance, Ramos-Garcia & Hoover [39] found that HMM outperforms KNN by approximately 13% when distinguishing between four activities (rest, bite, drink, using utensils). Further, they found that taking into account inter-gesture sequential dependencies further improves model performance (up to 96.5% accuracy). Kyritsis and colleagues [61] showed that replacing HMM with RNN improves the performance of the model even more. Taken together, these results hint at the importance of utilising the sequential context.

Notably, up to 2017, there was no study that utilised deep learning to detect eating behaviour in this context. Driven by the growing computing power, and specifically the availability of GPU-based high-performance computing, researchers increasingly explore the application of deep networks such as CNN and RNN (specifically Long Short-Term Memory networks, LSTM) to various classification problems (e.g., since 2010 in human affect recognition [82]). Since 2017, five studies have investigated the application of deep learning for assessing eating behaviour based on movement sensors ([61,68,71,73,75]). Results show that in an end-to-end deep learning solution a combination of CNN and RNN performs significantly better than a CNN-only solution while the models have no knowledge of micro-movements, also known as sub-gestures [73]. This will also simplify the annotation process since less detailed labelling regime will be required. As another example, Papadopoulos and colleagues [75] showed how an eating detection dataset can be used to (pre)train a LSTM and then fine-tune it on unlabelled data to adapt to a new participant using semi-supervised approved, allowing for a more personalised approach. Another application of deep learning is sensor fusion.

### 4.4. Public Database Development

Deep learning may not have been applied earlier in the eating behaviour context due to the inherent need for large datasets to train deep networks. Notably, compared to other domains such as object and human affect (e.g., face) recognition, there are few publicly available eating behaviour datasets with the total number of observations being relatively small (e.g., compared to affective computing where public datasets with millions of records exist; [83]). A related problem is that in order to accurately compare the performance of different classifiers, the models need to be evaluated using the same data. Hence, collecting and publishing reusable datasets can help researchers to compare the accuracy of models implemented based on different detection approaches. In recent years a few databases have been made public. In 2015, Thomaz and colleagues [2] published a lab and two free-living datasets (20 lab participants, seven free-living participants, one longitudinal free-living participant; http://www.ethomaz.com). In 2016, Mirtchouk and colleagues [48] published a wrist motion and audio sensors dataset (six participants; http://www.skleinberg.org/data.html). In 2017, Kyritsis and colleagues [61] published a food intake cycle dataset (10 participants; https://mug.ee.auth.gr/intake-cycle-detection). Finally, in 2018, Shen and colleagues [77] published a dataset that consists of 51,614 manually labelled gestures from 169 participants that was developed over the course of several studies (http: //cecas.clemson.edu/∼ahoover/cafeteria). This highlights the considerable amount of time and effort to prepare such a dataset. The growing availability of such datasets will help advance training classifiers in this area. In particular, publicly available datasets can provide the opportunity to pre-train models that can then be enhanced and improved on for specific hand gestures, or for a specific participant [75]. Further, this will allow better comparison and reconciliation of different ways of annotating eating gestures, which in turn facilitates enhanced comparison of the accuracy achieved across different types of sensors and algorithms.

### 4.5. Granularity of Eating Behaviour Detection and Sensor Fusion

In the context of dataset availability, it is noteworthy that the majority of studies, and especially those published in earlier calendar years, exclusively focus on a binary detection in terms of eating versus non-eating; both in terms of detecting overall eating occasions as well as individual hand gestures. While this binary classification provides a range of interesting insights (e.g., in terms of identifying the time, duration, and speed of eating), it does not consider other important aspects of eating such as the type (e.g., rice vs noodle [38]; distinguishing different drinks [64]) and amount of food being consumed (e.g., drink volume [64]), the category of eating utensil and serving vessel used (e.g., distinguishing chopsticks, hand, and spoon [35]), or related hand gestures (e.g., using cutlery to prepare food items for intake, using spoon to transfer food into serving vessel). Over time, the binary detection of eating occasions and individual hand-to-mouth movements has improved substantially. However, improving the detection of eating utensils and the amount of food that is being consumed will require more sophisticated models, larger reference datasets, and synthesis with established dietary assessment tools. Image-based food records [84] are well suited to complement data capture of hand-to-mouth movement data, due to the collection of type and amount of food, in addition to timing, and are preferred to traditional methods such as weighed food records [85]. Leveraging the potential of automating model configuration and employing end-to-end models that require less detailed annotations could be important steps in this direction.

In terms of sensor fusion, studies combined (1) different kinds of motion sensors (e.g., accelerometer, gyroscope, magnetic coupling and RFID sensors), (2) upper limb-mounted motion sensors with motion sensors mounted to other body parts (e.g., torso, jaw; [37]), and (3) motion sensors with other different types of sensors (e.g., camera, microphone, scales). Particularly when non-motion sensors are used, the goal is usually to narrow down the location (e.g., which room in smart homes, [31]) or activity of the user and, hence, reduce or remove confounding gesture types in free-living settings. Further, in earlier studies, some primarily focused on accelerometers because at that time gyroscopes required considerable amounts of energy. However, with the recent advances in gyroscope and battery technologies, these obstacles have been overcome for most settings. Further, in an effort to save energy, some studies used a hybrid approach where the gyroscope was only activated when the accelerometer detected a series of eating associated gestures [70]. A similar approach was used to start recordings with wearable cameras [7]. Shibon and Amft [76] applied a controller to the sensing and processing system to increase the sample and processing rate once a rotational hand gesture is detected. Hence, despite the progress in technology, these approaches might still be useful in scenarios where access to power is limited (e.g., in low and lower-middle income country settings) or where motion data is to be complemented with energy or storage intensive video recordings. However, concerns on privacy of wearable cameras need to be acknowledged, and the impact on behaviours relating to eating has not been determined. Alternatively, active image capture methods, such as image-based food records collected via mobile devices [84], allow for collection of data on food type and amount, meal composition and temporal eating patterns which could be combined with wrist motion sensor data in new ways such as to verify intake data from such self-reported tools.

### 4.6. Applicability in Dietary Assessment and Eating Behaviour Interventions

While initially, studies relied on specialised research equipment or dedicated hardware prototypes, recent advances in accuracy and affordability of wearable sensing technology have made commercial-grade sensors widely accessible. Increasingly, studies rely on off-the-shelf devices such as smart watches, demonstrating that such devices are considered reliable and accurate for detecting eating behaviour (e.g., [61,62,67]). This has important implications for the real-world feasibility of using this technology for dietary assessment and monitoring [86]. In particular, because watches have been worn on the wrist for more than a century, using wearable sensors on the wrist is an unobtrusive solution for collecting movement data. Hence, readily available smartwatches could provide the infrastructure to implement end-user applications that allow to track eating behaviour (e.g., [86,87]). However, the software infrastructure is yet to be developed to collect, store, and analyse personal data. For instance, the computing power of smart watches could be used for an online detection of eating behaviour and the delivery of context-sensitive behavioural recommendations. Further, by establishing a data exchange with health practitioners and others, such systems could provide targeted recommendations that promote positive health outcomes [88]. In the case of disease management, for instance, this data could be used by health practitioners to keep track of a patient’s dietary intake behaviour and characteristics and provide them with useful dietary advice.

### 4.7. Strengths and Limitations of the Current Review

The current review has strengths and limitations that should be considered in the interpretation of its findings. A strength is that it is the first systematic review on the automatic detection of eating behaviour based on upper limb-mounted motion sensors following a rigorous review approach. Based on this, this review provides the first comprehensive overview of study settings, sensor configurations, action classes, performance comparisons, and detection approaches for assessing eating behaviour from upper limb motion sensors. The developed framework conceptualises the components and implicit design choices that researchers and practitioners need to consider when carrying out studies and may hence facilitate further research in this area. Further, by searching across 11 different databases, we cover health and dietary assessment journals as well as computing-focused ones. Nevertheless, it needs to be acknowledged that only considering studies published in English language may constitute a limitation. Further, due to the limitation of number of search terms, our search string only covers plural forms for word combinations. This is based on the advice of a medical librarian we consulted with that search databases will automatically detect plural forms for single terms (e.g., “smartphone” will cover “smartphones”) but not for word combinations (e.g., “arm movement” will not cover “arm movements”). Finally, focusing only on upper limb-mounted wrist sensors does not take into account other sensor positions (e.g., head, neck) and associated sensor fusion approaches (e.g., microphone).

## 5. Conclusions

To date, 69 studies have investigated upper limb-mounted motion sensors for automatic eating behaviour recognition. These studies were predominantly laboratory based and were undertaken by university students, employed shallow machine learning architectures, and focused on distinguishing eating from non-eating activities. At this stage, five studies have successfully employed deep learning architectures in this context. The availability of large public databases will be paramount to progressing the development of more fine-grained eating behaviour assessment approaches. This will allow future research to directly compare the accuracy of different classifiers, consider multiple contextual factors inherent to eating (e.g., communal eating, culture), and to transfer those models from controlled laboratory conditions to practical free-living settings in different countries (e.g., low and lower-middle income) and eating contexts (e.g., home vs work environment, social gatherings).

## Figures and Tables

**Table 1:** Terms used in this review with synonyms and definitions.

Term	Synonyms Used in the Literature	Definition
**Action classes**	Event/activity classifications/categories	Different categories that the classifiers (detection models) are trained and tested to classify
**Artificial intelligence model**	Artificial intelligence approach, machine learning algorithm	The approach used for automatic eating behaviour detection
**Backward search**		Search through references of included studies to find other relevant studies that are not found through database search
**Eating activity**		Eating and drinking activity
**Eating behaviour assessment**	Food intake detection, eating detection, ingestion monitoring	Assessing whether the participant is eating (including drinking) and what their eating characteristics are
**Forward search**		Search for relevant studies that cited included studies
**F-score**	F1 score, F-measure	F-score is a measure of accuracy. While accuracy is the total number of correctly classified items divided by all classified items, F-score is harmonic average of the precision and recall.
**Hand-to-mouth gesture**	Hand-to-mouth movement	The movement of hand carrying food with or without utensils to the mouth
**Motion sensors**	Motion tracking sensors, motion detection sensors, activity tracker	Sensors used to detect movements. Wearable motion sensors focused on in the current review include upper limb-mounted motion sensors.
**Participant**	Subject	An individual who has successfully participated in a study (i.e., not counting individuals who were invited but did not participate or individuals with failed measurements)
**Upper limb**	Arm	Region of body that includes shoulder, upper arm, lower arm, wrist, and hand

**Table 2:** Included studies (*n* = 69).

Author (Year), Country [Ref]	Type	Participants (Environment)	Upper Limb Sensor (Frequency) [Position]	Comparator	Algorithm (Detection)
Amft et al. (2005), Switzerland [23]	Conference	2 (lab)	Acc/Gyro (100 Hz) [wrist, both]	NR	FSS, HMM (GD)
Amft et al. (2007), Switzerland [24]	Conference	1 (lab)	Acc/Gyro (NR) [lower arm, both]	NR	PCFG (AD)
Amft et al. (2008), Switzerland [25]	Journal	4 (lab)	Acc/Gyro (100 Hz) [lower arm, both]	NR	FSS (GD)
Junker et al. (2008), Switzerland [26]	Journal	4 (Lab)	Acc/Gyro (100 Hz) [lower arm, both]	NR	HMM (GD)
Laerhoven et al. (2008), Germany [27]	Conference	2 (free-living)	Acc (NR) [wrist, dominant]	Other self-report	KNN (AD)
Pirkl et al. (2008), Germany [28]	Conference	1 (lab)	Acc/Gyro (50 Hz), Prox [lower arm, dominant]	NR	DT (GD)
Tolstikov et al. (2008), Singapore [29]	Conference	NR (lab)	Acc (50 Hz), Prox [wrist, dominant]	Camera	DBN (AD)
Zhang et al. (2008), Singapore [30]	Conference	NR (lab)	Acc (NR) [wrist, both]	NR	HTM (GD)
Dong et al. (2009), USA [13]	Conference	10 (lab)	Acc/Gyro (60 Hz) [wrist, dominant]	Camera	C/RB (GD)
Teixeira et al. (2009), USA [31]	Conference	1 (free-living)	Acc/Gyro (NR) [wrist, dominant]	NR	FSM (AD)
Amft et al. (2010), Netherlands [32]	Conference	9 (lab)	Acc/Gyro (87 Hz), Prox [wrist, dominant]	Camera	DT, FSS (GD)
Dong et al. (2011), USA [33]	Conference	4 (free-living)	Acc/Gyro (60 Hz) [wrist, dominant]	Diary	C/RB (AD)
Dong et al. (2012), USA [3]	Journal	102 (both)	Acc/Gyro (NR) [wrist, dominant]	Camera, Diary	C/RB (GD)
Grosse-Puppendahl et al. (2012), Germany [34]	Chapter	7 (lab)	Acc (NR), Prox (NR) [wrist, dominant]	NR	SVM (AD)
Kim et al. (2012), South Korea [35]	Conference	13 (lab)	Acc (30 Hz) [wrist, dominant]	Camera	DT (CD, GD)
Mousavi Hondori et al. (2012), USA [36]	Conference	1 (lab)	Acc (NR) [utensil, both]	NR	NR (NR)
Varkey et al. (2012), USA [8]	Journal	1 (lab)	Acc/Gyro (20 Hz) [wrist, dominant]	NR	SVM (AD)
Farooq et al. (2013), USA [5]	Conference	13 (free-living)	Prox (NR) [wrist, dominant]	Diary, Push Button	ANN, SVM (AD)
Fontana et al. (2013), USA [37]	Conference	12 (free-living)	Prox (10 Hz) [wrist, dominant]	Push Button	RF (AD)
Kim & Choi (2013), South Korea [38]	Conference	8 (lab)	Acc (30 Hz) [wrist, dominant]	Camera	DT, NB (AD, CD, GD)
Ramos-Garcia & Hoover (2013), USA [39]	Conference	273 (lab)	Acc/Gyro (15 Hz) [wrist, dominant]	Camera	HMM, KNN (GD)
Desendorf et al. (2014), USA [40]	Journal	15 (lab)	Acc (80 Hz) [wrist, dominant]	NR	C/RB (GD)
Dong et al. (2014), USA [41]	Journal	43 (free-living)	Acc/Gyro (NR) [lower arm, dominant]	Mobile App	C/RB, NB (GD, AD)
Fontana et al. (2014), USA [6]	Journal	12 (free-living)	Prox (NR) [wrist, dominant]	Mobile App, Push Button	ANN (AD)
Ramos-Garcia et al. (2015), USA [12]	Journal	25 (lab)	Acc/Gyro (15 Hz) [wrist, dominant]	Camera	HMM, KNN (GD)
Sen et al. (2015), Singapore [42]	Conference	6 (lab)	Acc/Gyro (100 Hz) [wrist, dominant]	Wearable Camera	C/RB (GD)
Thomaz et al. (2015), USA [2]	Conference	28 (both)	Acc (25 Hz) [wrist, dominant]	Camera, Wearable Camera	DBSCAN, KNN, RF, SVM (AD, GD)
Ye et al. (2015), USA [43]	Conference	10 (lab)	Acc (50 Hz) [wrist, dominant]	Camera	DT, NB, SVM (GD)
Zhou et al. (2015), Japan [9]	Journal	5 (lab)	Acc (NR) [finger, dominant]	NR	DT, KNN (GAD)
Fan et al. (2016), USA [44]	Journal	1 (lab)	Acc/Gyro (50.1 Hz) [wrist, dominant]	NR	ANN, DT, KNN, NB, Reg, RF, SVM (AD)
Farooq & Sazonov (2016), USA [45]	Conference	12 (free-living)	Prox. (NR) [wrist, dominant]	Mobile App, Push Button	DT, Reg (AD)
Fortuna et al. (2016), USA [46]	Conference	3 (free-living)	Acc/Gyro (10 Hz) [wrist, dominant]	Camera, Wearable Camera	NB (GD)
Kim et al. (2016), South Korea [4]	Conference	15 (lab)	Acc/Gyro (NR) [wrist, dominant]	Camera	C/RB (GD)
Maramis et al. (2016), Greece [47]	Conference	8 (lab)	Acc/Gyro (NR) [wrist, dominant]	Camera	SVM (GD)
Mirtchouk M. et al. (2016), USA [48]	Conference	6 (lab)	Acc/Gyro (15 Hz) [wrist, both]	Camera	RF (CD)
Parra-Sanchez et al. (2016), Mexico [49]	Conference	7 (lab)	Electro-hydraulic (NR) [arm, both]	Camera	DT, HMM (GAD)
Rahman et al. (2016), Canada [50]	Conference	8 (free-living)	Acc/Gyro (15 Hz) [wrist, dominant]	Mobile App	DT, Reg, RF, SVM (AD, CD)
Sharma et al. (2016), USA [51]	Conference	94 (free-living)	Acc/Gyro (15 Hz) [wrist, dominant]	NR	C/RB, NB (AD, GD)
Shen et al. (2016), USA [52]	Conference	215 (lab)	Acc/Gyro (15 Hz) [wrist, dominant]	Camera	HMM (GD)
Shoaib et al. (2016), Netherlands [53]	Conference	11 (lab)	Acc/Gyro (50 Hz) [wrist, dominant]	NR	DT, RF, SVM (AD)
Ye et al. (2016), USA [54]	Conference	7 (free-living)	Acc (50 Hz) [wrist, dominant]	Mobile App	SVM (GD)
Zhang et al. (2016), USA [55]	Conference	15 (lab)	Acc/Gyro (31 Hz) [wrist, both]	Camera	DBSCAN, DT, NB, Reg, RF, SVM (GD, AD)
Alexander et al. (2017), USA [56]	Journal	4 (lab)	Acc/Gyro (20 Hz) [wrist, dominant]	Time Sync	NB (AD)
Bi et al. (2017), USA [57]	Journal	37 (free-living)	Acc (80 Hz) [wrist, dominant]	Other self-report	HMM, SVM (AD)
Dong & Biswas (2017), USA [14]	Journal	14 (lab)	Acc (100 Hz) [wrist, dominant]	Camera, Push Button	C/RB, HMM, SVM (GD, AD)
Egilmez et al. (2017), USA [58]	Journal	9 (lab)	Acc/Gyro (5 Hz) [wrist, dominant]	NR	NB, Reg, RF, SVM (GAD)
Garcia-Ceja et al. (2017), Mexico [59]	Journal	3 (lab)	Acc/Gyro (31 Hz) [wrist, dominant]	NR	RF (GAD)
Kyritsis et al. (2017), Greece [60]	Conference	8 (lab)	Acc/Gyro (62 Hz) [wrist, dominant]	Camera	HMM, SVM (AD, GD)
Kyritsis et al. (2017), Greece [61]	Conference	10 (lab)	Acc/Gyro (62 Hz) [wrist, dominant]	Camera	DL, SVM (AD, GD)
Loke & Abkenar (2017), Australia [62]	Journal	4 (lab)	Acc (NR) [wrist, dominant]	NR	DT (GAD)
Moschetti et al. (2017), Italy [63]	Conference	12 (lab)	Acc/Gyro (50 Hz) [wrist, dominant]	NR	GMM, KM, RF, SVM (GD)
Sen et al. (2017), Singapore [7]	Journal	28 (both)	Acc/Gyro (NR) [wrist, dominant]	Camera, Diary	DT, RF, SVM (AD, GD)
Shen et al. (2017), USA [1]	Journal	271 (lab)	Acc/Gyro (15 Hz) [wrist, dominant]	Camera	C/RB (GD)
Soubam et al. (2017), India [64]	Conference	11 (lab)	Acc (186 Hz) [wrist, dominant]	Time Sync	DT, NB, RF, SVM (CD, GD)
Thomaz et al. (2017), USA [65]	Conference	14 (lab)	Acc/Gyro (30 Hz) [wrist, both]	Camera	RF (AD)
Yoneda & Weiss (2017), USA [66]	Conference	51 (lab)	Acc/Gyro (20 Hz) [wrist, dominant]	NR	DT, KNN, RF (GAD)
Zhang et al. (2017), USA [67]	Conference	8 (free-living)	Acc/Gyro (31 Hz) [wrist, both]	Wearable Camera	RF (GD)
Anderez et al. (2018), UK [68]	Conference	NR (lab)	Acc/Gyro (100 Hz) [wrist, dominant]	NR	DL, RF (GD)
Anderez et al. (2018), UK [69]	Conference	NR (lab)	Acc (100 Hz) [wrist, dominant]	NR	KNN (GD)
Balaji et al. (2018), India [70]	Conference	NR (NR)	Acc/Gyro (100 Hz) [wrist, NR]	NR	C/RB (GAD)
Cho & Choi (2018), South Korea [71]	Conference	8 (lab)	Acc (50 Hz) [wrist, dominant]	Camera	DL (CD, GD)
Clapés et al. (2018), Spain [72]	Journal	14 (lab)	Acc/Gyro (25 Hz) [wrist, dominant]	Camera	ANN, Opt (GAD, GD)
Kyritsis et al. (2018), Greece [73]	Conference	10 (lab)	Acc/Gyro (100 Hz) [wrist, dominant]	Camera	DL (AD)
Manzi et al. (2018), Italy [74]	Journal	20 (lab)	Acc/Gyro (NR) [wrist, dominant]	NR	RF (GAD)
Papadopoulos et al. (2018), Greece [75]	Conference	10 (lab)	Acc/Gyro (62 Hz) [wrist, dominant]	Camera	semi-supervised DL (AD)
Schibon & Amft (2018), Germany [76]	Conference	6 (free-living)	Acc/Gyro (NR) [wrist, both]	Diary	SVM (GD)
Shen et al. (2018), USA [77]	arXiv	269 (lab)	Acc/Gyro (15 Hz) [wrist, dominant]	Camera	HMM (GD)
Zambrana et al. (2018), Spain [78]	Journal	21 (lab)	Acc (20 Hz) [wrist, both]	Camera	KNN, RF, SVM (GAD)
Zhang et al. (2018), USA [17]	Journal	10 (lab)	Acc/Gyro (NR) [wrist, dominant]	Camera	RF (GD)

*Note:* The table only includes performance comparisons where studies have directly compared different algorithms using the same dataset. Acc = Accelerometer, AD = Eating Activity Detection, ANN = Artificial Neural Network, CD = Eating Characteristics Detection, Chap = Book Chapter, Conf = Conference, C/RB = Custom Rule-Based, DBN = Dynamic Bayesian Network, DBSCAN = Density-Based Spatial Clustering of Applications with Noise, DL = Deep Learning, DT = Decision Tree, FL = Free-Living, FSM = Finite State Machine, FSS = Feature Similarity Detection, GAD = General Activity Detection, GD = Eating Gesture Detection, GMM = Gaussian Mixture Model, Gyro = Gyroscope, HMM = Hidden Markov Model, HTM = Hierarchical Temporal Memory, Jour = Journal, KM = K-Means, KNN = K-Nearest Neighbours, NB = Naive Bayes, NR = Not Reported, Opt = Monte Carlo Optimization method, PCFG = Probabilistic Context-Free Grammar, Prox = Proximity, Reg = Regression, RF = Random Forest, SVM = Support Vector Machine.

**Table 3:** Machine learning algorithms used in the included studies as well as and detection approaches and performance comparisons conducted in the studies.

Algorithm (# and % Studies)—Approach	Best Performing Algorithm (vs. Comparison Algorithm/s)	Performance Comparison Results
**SVM (21, 30.4%)** AD: [5,8,34,44,53,57], AD/CD: [50], CD/GD: [64], GAD: [58,78], GD: [43,47,54,63,76], GD/AD: [2,7,14,55,60,61]	SVM (vs. DT, NB) [43] (GD)	Using only wrist or head motion data SVM accuracy for eating detection was between 0.895 to 0.951 (combined wrist/head: 0.970).
	SVM (vs. KNN, RF) [78] (GAD)	Best accuracy of SVM using time-domain features was 0.957 (F = 0.957, two-second window). Best accuracy of RF model using frequency-domain features was 0.939 (F = 0.940, four-second window).
	SVM (vs. DT, RF) [53] (AD)	F-scores for detecting eating, drinking, and smoking with SVM were 0.910, 0.780, and 0.830, compared to RF with 0.860, 0.780, and 0.840, and DT with 0.820, 0.690, and 0.780.
**RF (19, 27.5%)** AD: [37,44,53,65], AD/CD: [50], CD: [48], CD/GD: [64], GAD: [58,59,66,74,78], GD: [17,63,67,68], GD/AD: [2,7,55]	RF (vs. SVM, KNN) [2] (GD)	FR outperformed SVM and 3-NN in detecting eating gestures in two free-living settings (seven participants in one day, F = 0.761; one participant in 31 days (F = 0.713).
	RF (vs. DT, NB, Reg, SVM) [55] (GD)	Eating gesture detection with RF yielded F = 0.753 compared to F = 0.708 (SVM), F = 0.694 (Reg), F = 0.647 (DT), and F = 0.634 (NB).
	RF (vs. DT, SVM) [7] (GD)	The accuracies achieved by RF, DT, and SVM were 0.982, 0.966, and 0.857, respectively.
	RF (vs. SVM) [63] (GD)	Using leave one person out cross-validation method the accuracies achieved by RF and SVM were 0.943 (F = 0.949) and 0.882 (F = 0.895).
	RF (vs. NB, Reg, SVM) [58] (GAD)	F-scores for general activity detection with FR, Reg, SVM, and NB were 0.788, 0.661, 0.6l3, and 0.268, respectively (eating was among action classes).
	RF (vs. DT, NB, SVM) [64] (GD)	The accuracies of RF, SVM, DT, and NB in person independent drinking versus eating detection were 0.924, 0.905, 0.881 and 0.871, respectively.
	RF (vs. DT, Reg, SVM) [50] (AD)	Person independent “about-to-eat” detection achieved F = 0.690 (RF) compared to 0.660 (SVM), 0.660 (REG), and 0.640 (DT).
	RF (vs. DT, KNN) [66] (GAD)	Accuracies of RF, DT, and KNN were 0.997, 0.980, and 0.888, respectively (using accelerometer data).
**DT (16; 23.2%)** AD: [44,45,53], AD/CD: [50], CD/GD: [35,64], CD/GD/AD: [38], GAD: [9,49,62,66], GD: [28,32,43], GD/AD: [7,55]	DT (vs. NB) [38] (AD/CD)	Best F-scores of DT and NB were 0.930 and 0.900 (eating activity detection) and 0.780 and 0.700 (eating type detection), respectively.
	DT (vs. NB) [35] (CD/GD)	Best F-scores of DT and NB were 0.750 and 0.650 (eating utensil detection) and 0.280 and 0.190 (eating action detection), respectively.
**HMM (10, 14.5%)** AD: [57], GAD: [49], GD: [12,23,26,39,52,77], GD/AD: [14,60]	HMM (vs. SVM) [57] (AD)	HMM outperformed SVM by 6.82% on recall in family meal detection, while the average precision and recall were 0.807 and 0.895.
	HMM (vs KNN) [39] (GD)	Accuracies of HMM, and KNN were 0.843 and 0.717, respectively.
	HMM-1 (vs. HMM-S, HMM-N, N:2-6) [77] (GD)	Accuracies of HMM-S and gesture-to-gesture HMM-1 were 0.852 and 0.895 respectively. According to the figure provided in the study the accuracy for HMM-2 to 4 stays similar and decreases for HMM-5 and 6.
	HMM-6 (vs. HMM-N, N:1-5, KNN, S-HMM) [12] (GD)	The accuracies of HMM-6, HMM-5, HMM-4, HMM-3, HMM-2, HMM-1, sub-gesture HMM and KNN were 0.965, 0.946, 0.922, 0.896, 0.880, 0.877, 0.843 and 0.758, respectively.
**KNN (9, 13%)** AD: [27,44], GAD: [9,66,78], GD: [12,39,69], GD/AD: [2]	KNN (vs. ANN, DT, NB, Reg, RF, SVM) [44] (AD)	The accuracies of KNN, RF, Reg, SVM, ANN, NB and DT were 0.936, 0.933, 0.923, 0.920, 0.913, 0.906 and 0.893, respectively.
	KNN (vs. DT, NB) [9] (GAD)	The precision (and recall) values of KNN, DT and NB were 0.710 (0.719), 0.670 (0.686) and 0.657 (0.635), respectively.
**DL (5, 7.2%)** AD: [73,75], CD/GD: [71], GD: [68], GD/AD: [61]	RNN (vs. HMM) [61,73] (AD)	Replacing HMM with RNN in a SVM-HMM model improved F-score from 0.814 to 0.892 [61]. In a subsequent study a single-step end-to-end RNN reached almost the same performance (F = 0.884) [73].
**ANN (4, 5.8%)** AD: [5,6,44], GD/GAD: [72]	ANN (vs. SVM) [5] (AD)	ANN achieved accuracy of 0.869 (±0.065) compared to SVM (0.819, ±0.092) for eating activity detection (12 participants). ANN achieved accuracy of 0.727 compared to SVM (0.636, ±0.092) for number of meals detection (1 participant).
**KM (1, 1.4%)** GD: [63]	KM (vs. GMM) [63] (GD)	In an inter-person comparison, the accuracies of unsupervised approaches KM and GMM were 0.917 (F = 0.920) and 0.796 (F = 0.805), respectively.
**Other:** C/RB (11, 15.9%, AD [33], GAD [70], GD [1,3,4,13,40,42], GD/AD [14,41,51]), NB (11, 15.9%, AD [44,56], CD/GD [35,64], CD/GD/AD [38], GAD [58], GD [43,46], GD/AD [41,51,55]), Reg (5, 7.2%, AD [44,45], AD/CD [50], GAD [58], GD/AD [55]), FSS (3, 4.3%, GD: [23,25,32]), DBSCAN (2, 2.9%, GD/AD [2,55]), DBN (1, 1.4%, AD [29]), FSM (1, 1.4%, AD [31]), HTM (1, 1.4%, GD [30]), Opt (1, 1.4%, GD/GAD [72]), PCFG (1, 1.4%, AD [24])		

*Note:* AD = Eating Activity Detection, ANN = Artificial Neural Network, CD = Eating Characteristics Detection, C/RB = Custom Rule-Based, DBN = Dynamic Bayesian Network, DBSCAN = Density-Based Spatial Clustering of Applications with Noise, DL = Deep Learning, DT = Decision Tree, F = F-score, FSM = Finite State Machine, FSS = Feature Similarity Detection, GAD = General Activity Detection, GD = Eating Gesture Detection, GMM = Gaussian Mixture Model, HMM = Hidden Markov Model, HMM-S = single-gesture HMM, HTM = Hierarchical Temporal Memory, KM = K-Means, KNN = K-Nearest Neighbours, NB = Naive Bayes, Opt = Monte Carlo Optimization method, PCFG = Probabilistic Context-Free Grammar, Reg = Regression, RF = Random Forest, RNN = Recurrent Neural Network, SVM = Support Vector Machine.

## Supplementary Materials

The following are available online at https://www.mdpi.com/2072-6643/11/5/1168/s1, Table S1: List of search strategy strings.

## Appendix A

**Table A1:** Search strategy string.

Search String
(accelerometer OR gyroscope OR smartwatch OR “inertial sensor” OR “inertial sensors” OR “inertial sensing” OR smartphone OR “cell phone” OR wristband) AND (“dietary intake” OR “dietary assessment” OR “food intake” OR “nutrition assessment” OR “eating activity” OR “eating activities” OR “eating behavior” OR “eating behaviour” OR “energy intake” OR “detecting eating” OR “detect eating” OR “eating episodes” OR “eating period”) AND (“bite counting” OR “counting bites” OR “hand gesture” OR “hand gestures” OR “arm gesture” OR “arm gestures” OR “wrist gesture” OR “wrist gestures” OR “hand motion” OR “hand motions” OR “arm motion” OR “arm motions” OR “wrist motion” OR “wrist motions” OR “hand movement” OR “hand movements” OR “arm movement” OR “arm movements” OR “wrist movement” OR “wrist movements” OR “hand to mouth” OR “hand-to-mouth” OR “wrist-worn” OR “wrist-mounted”)

**Table A2:** Search strategy databases (English only results).

Database name	Result
ACM	10
AIS Electronic Library	55
CINAHL	16
EMBASE ^1^	
IEEE ^2^	140
MEDLINE ^1^	
Ovid databases ^3^	54
ScienceDirect ^4^	133
Scopus	161
SpringerLink	205
Web of Science	18
Total results before removing duplicates	792
Total results after removing duplicates	653

^1^ Results from Ovid databases include results from databases EMBASE and MEDLINE. ^2^ Due to limitation on the length of the search string that IEEE database accepted, the search string was broken up to smaller parts (see Supplementary Material). ^3^ Ovid databases include Books@Ovid, Embase, Emcare, MEDLINE. The following option were chosen to be included in the results: AMED (Allied and Complementary Medicine) 1985 to September 2017, Books@Ovid September 25, 2017, Embase 1947 to present, Emcare (Nursing and Allied Health) 1995–present, International Pharmaceutical Abstracts 1970 to September 2017, Medline 1946–present, University of Newcastle Journals. ^4^ ScienceDirect database web search did not accept the search terms “hand-to-mouth” and “hand to mouth”. Therefore, these two search terms were excluded from the search string submitted to ScienceDirect. Also due to limitation on the length of the search string that ScienceDirect database accepted, the search string was broken up to smaller parts (see Supplementary Material).
