# Supplementary material for: Assessing Eating Behaviour Using Upper Limb Mounted Motion Sensors: A Systematic Review
Source: Nutrients. 2019 May 24;11(5):1168. doi: 10.3390/nu11051168 (PMC6566929; doi:10.3390/nu11051168)
Supplement: Supplementary file 1 [file nutrients-11-01168-s001.pdf]

nutrients

MDPI

Supplementary Material

Search Strategy

The main search string was used on ACM, AIS Electronic Library, CINAHL, EMBASE, MEDLINE, Ovid databases, Scopus databases, and SpringerLink. ScienceDirect database did not accept the search terms "hand-to-mouth" and "hand to mouth". Therefore, these two search terms were removed from the search string submitted to ScienceDirect. Due to limitation on the length of the search string that ScienceDirect database accepted, the search string was broken up to ScienceDirect Search Sub String #1, #2, #3 and #4. Due to limitation on the length of the search string that IEEE database accepted, the search string for IEEE database was broken up to IEEE Search Sub String #1, #2, #3 and #4. Table 1 provides the search strings submitted to the search databases.

Table 1. List of search strategy strings

Search string name	Search string
**Main Search String**	(accelerometer OR gyroscope OR smartwatch OR "inertial sensor" OR "inertial sensors" OR "Inertial sensing" OR smartphone OR "cell phone" OR wristband) AND ("dietary intake" OR "dietary assessment" OR "food intake" OR "nutrition assessment" OR "eating activity" OR "eating activities" OR "eating behavior" OR "eating behaviour" OR "Energy intake" OR "detecting eating" OR "detect eating" OR "eating episodes" OR "eating period") AND ("bite counting" OR "counting bites" OR "hand gesture" OR "hand gestures" OR "arm gesture" OR "arm gestures" OR "wrist gesture" OR "wrist gestures" OR "hand motion" OR "hand motions" OR "arm motion" OR "arm motions" OR "wrist motion" OR "wrist motions" OR "hand movement" OR "hand movements" OR "arm movement" OR "arm movements" OR "wrist movement" OR "wrist movements" OR "hand to mouth" OR "hand-to-mouth" OR "wrist-worn" OR "wrist-mounted")
**ScienceDirect Search Sub String #1**	(accelerometer OR gyroscope OR smartwatch OR "inertial sensor" OR "inertial sensors" OR "Inertial sensing" OR smartphone OR "cell phone" OR wristband) AND ("dietary intake" OR "dietary assessment" OR "food intake" OR "nutrition assessment" OR "eating activity" OR "eating activities" OR "eating behavior" OR "eating behaviour" OR "Energy intake" OR "detecting eating" OR "detect eating" OR "eating episodes" OR "eating period") AND ("bite counting" OR "counting bites" OR "hand gesture" OR "hand gestures" OR "arm gesture" OR "arm gestures")
**ScienceDirect Search Sub String #2**	(accelerometer OR gyroscope OR smartwatch OR "inertial sensor" OR "inertial sensors" OR "Inertial sensing" OR smartphone OR "cell phone" OR wristband) AND ("dietary intake" OR "dietary assessment" OR "food intake" OR "nutrition assessment" OR "eating activity" OR "eating activities" OR "eating behavior" OR "eating behaviour" OR "Energy intake" OR "detecting eating" OR "detect eating" OR "eating episodes" OR "eating period") AND ("wrist gesture" OR "wrist gestures" OR "wrist motion" OR "wrist motions" OR "wrist movement" OR "wrist movements" OR "wrist-worn" OR "wrist-mounted")
**ScienceDirect Search Sub String #3**	(accelerometer OR gyroscope OR smartwatch OR "inertial sensor" OR "inertial sensors" OR "Inertial sensing" OR smartphone OR "cell phone" OR wristband) AND ("dietary intake" OR "dietary assessment" OR "food intake" OR "nutrition assessment" OR "eating activity" OR "eating activities" OR "eating behavior" OR "eating behaviour" OR "Energy intake" OR "detecting

	eating" OR "detect eating" OR "eating episodes" OR "eating period") AND ("hand motion" OR "hand motions" OR "arm motion" OR "arm motions" OR "arm movement" OR "arm movements")
**ScienceDirect Search Sub String #4**	(accelerometer OR gyroscope OR smartwatch OR "inertial sensor" OR "inertial sensors" OR "Inertial sensing" OR smartphone OR "cell phone" OR wristband) AND ("dietary intake" OR "dietary assessment" OR "food intake" OR "nutrition assessment" OR "eating activity" OR "eating activities" OR "eating behavior" OR "eating behaviour" OR "Energy intake" OR "detecting eating" OR "detect eating" OR "eating episodes" OR "eating period") AND ("hand movement" OR "hand movements")
**IEEE Search Sub String #1**	accelerometer OR gyroscope OR smartwatch OR "inertial sensor" OR "inertial sensors" OR "Inertial sensing" OR smartphone OR "cell phone" OR wristband
**IEEE Search Sub String #2**	"dietary intake" OR "dietary assessment" OR "food intake" OR "nutrition assessment" OR "eating activity" OR "eating activities" OR "eating behavior" OR "eating behaviour" OR "Energy intake" OR "detecting eating" OR "detect eating" OR "eating episodes" OR "eating period"
**IEEE Search Sub String #3**	"hand gesture" OR "hand gestures" OR "arm gesture" OR "arm gestures" OR "hand motion" OR "hand motions" OR "arm motion" OR "arm motions" OR "hand movement" OR "hand movements" OR "arm movement" OR "arm movements"
**IEEE Search Sub String #4**	"bite counting" OR "counting bites" OR "wrist gesture" OR "wrist gestures" OR "wrist motion" OR "wrist motions" OR "wrist movement" OR "wrist movements" OR "hand to mouth" OR "hand-to-mouth" OR "wrist-worn" OR "wrist-mounted"

© 2018 by the authors. Submitted for possible open access publication under the terms and conditions of the Creative Commons Attribution (CC BY) license (http://creativecommons.org/licenses/by/4.0/).
